# Role of mechano-sensitive non-coding RNAs in bone remodeling of orthodontic tooth movement: recent advances

**DOI:** 10.1186/s40510-022-00450-3

**Published:** 2022-12-30

**Authors:** Lichao Yan, Li Liao, Xiaoxia Su

**Affiliations:** State Key Laboratory of Oral Diseases and National Clinical Research Center for Oral Diseases and Department of Pediatric Dentistry and Engineering Research Center of Oral Translational Medicine and National Engineering Laboratory for Oral Regenerative Medicine, West China Hospital of Stomatology, Sichuan University, Chengdu, 610041 China

**Keywords:** Bone remodeling, Biomechanics, Non-coding RNA, Periodontal tissue, Signaling regulation

## Abstract

Orthodontic tooth movement relies on bone remodeling and periodontal tissue regeneration in response to the complicated mechanical cues on the compressive and tensive side. In general, mechanical stimulus regulates the expression of mechano-sensitive coding and non-coding genes, which in turn affects how cells are involved in bone remodeling. Growing numbers of non-coding RNAs, particularly mechano-sensitive non-coding RNA, have been verified to be essential for the regulation of osteogenesis and osteoclastogenesis and have revealed how they interact with signaling molecules to do so. This review summarizes recent findings of non-coding RNAs, including microRNAs and long non-coding RNAs, as crucial regulators of gene expression responding to mechanical stimulation, and outlines their roles in bone deposition and resorption. We focused on multiple mechano-sensitive miRNAs such as miR-21, - 29, -34, -103, -494-3p, -1246, -138-5p, -503-5p, and -3198 that play a critical role in osteogenesis function and bone resorption. The emerging roles of force-dependent regulation of lncRNAs in bone remodeling are also discussed extensively. We summarized mechano-sensitive lncRNA XIST, H19, and MALAT1 along with other lncRNAs involved in osteogenesis and osteoclastogenesis. Ultimately, we look forward to the prospects of the novel application of non-coding RNAs as potential therapeutics for tooth movement and periodontal tissue regeneration.

## Introduction

Orthodontic tooth movement (OTM), the essential process to correct malocclusion, is recognized as a series of complex biological responses consisting of bone remodeling and periodontal tissue regeneration triggered by mechanical force (MF) [1, 2]. Characterized by the initial development of osteoblast (OB), osteoclast (OC), and their precursor cells, the process of OTM has been observed that the compressed and disorganized periodontal ligament (PDL) induces bone resorption, whereas the stretched PDL fibers lead to bone deposition [1]. Mechanical stimuli have consistently been proven to be crucial for sustaining bone metabolism and regulating osteogenic differentiation and bone formation [3]. OTM involves a variety of MFs in vitro, such as stretch stress, compressive force, and shear stress formed by fluid flow [4]. Here, we focus on the stretch stress and compressive force since they represent two major kinds of force and have been proven to determine the speed of OTM.

Periodontal ligament stem cells (PDLSCs) [5, 6], osteoblastic [7] cells, and osteocytes [8] are mechano-sensitive cells, which respond to orthodontic force primarily through numerous mechano-sensors presenting on the cell surface. These cells initiate inside-out or outside-in mechano-transduction via a variety of mechano-receptors, including extracellular matrix (ECM) molecules, transmembrane proteins, lipid bilayer, cytoskeleton, and nucleus. The mechanical signals activate downstream genes via various signal transmission pathways, leading to corresponding cellular effects. These force-altered genes are referred to as mechano-sensitive genes. Moreover, the majority of these mechano-sensitive genes are protein-coding genes, involving runt-related transcription factor 2 (RUNX2), β-catenin, and transcription factor Sp7 (also known as osterix) and osteocalcin (OCN), which are upregulated under the cyclic tensile stress [9–11]. The compressive force also upregulates several osteoclast-induced genes, including RANKL [12] and OC-STAMP [13], which mediate the proliferation and differentiation of OCs.

Non-coding RNA (ncRNA) is referred to as a type of RNA that does not translate into a protein. Abundant and functionally significant types of ncRNA include microRNAs (miRNAs), the long ncRNAs (lncRNAs), transfer RNAs (tRNAs), and ribosomal RNAs (rRNAs) et al. NcRNA accounts for approximately 98 percent of the entire genome [14] and regulates a myriad of physiological processes, involving cancer, Alzheimer’s disease, and periodontitis, via controlling the transcription, post-transcriptional, and epigenetic aspects of gene expression. Emerging evidence indicates that a larger cluster of ncRNA responds to the alteration of mechanical conditions both in vitro and in vivo. Further research revealed that these ncRNAs, known as mechano-sensitive ncRNAs, function as the cellular regulators that initially respond to mechanical signals and are essential for alveolar bone remodeling.

However, it remains to elucidate the role of these ncRNA on complex processes of OTM and the detailed mechanisms of mechanical stimuli to regulate the proliferation and differentiation of OB and OC via ncRNA. In this review, we will comprehensively discuss two main groups of ncRNAs: miRNAs (< 30 nucleotides) and lncRNAs (> 200 nucleotides). Additionally, we aim to identify the broad networks of ncRNA expression during force-induced bone remodeling and update the current understanding of the sequential expression profiles of ncRNA in OTM and their relevant therapeutic implications.

## Material and methods

### Protocol

The PRISMA was followed when this review’s literature was screened [15]. This protocol was not registered previously.

### Eligibility criteria

Articles, reviews, editorials, research letters, and systematic reviews that covered all of the subjects we included in the review, such as miRNA, lncRNA, and biomechanical and biological processes during OTM, all met the inclusion criteria for this review. Case reports, abstracts, and studies that did not involve the ncRNA and OTM mechanisms were omitted.

### Information sources

The following four databases were thoroughly searched to find all pertinent studies: MEDLINE (via PubMed), Embase, the Cochrane Library, and Web of Science. In addition, a manual search was done by looking through the associated articles’ reference lists. All searches were conducted in November 2021.

### Search

“RNA, Untranslated,” “bone remodeling,” “orthodontic tooth movement,” and “Biomechanical Phenomena” were the four keywords used in the search approach. The keyword “RNA, Untranslated” was expanded to “noncoding RNA” and “non-coding RNA,” and the abbreviation “ncRNA” was also concerned. The terms “orthodontic tooth movement,” “orthodontics,” “orthodontically,” and the acronym “OTM” were added to the phrase. The search strategy was as follows: “(“RNA, Untranslated”[Mesh]) and ((bone remodeling) or (orthodontic tooth movement) or (OTM))” and “(“RNA, Untranslated”[Mesh]) and (“Biomechanical Phenomena”[Mesh]),” which was developed for MEDLINE and modified for the other databases.

### Study selection

**Fig. 1:**
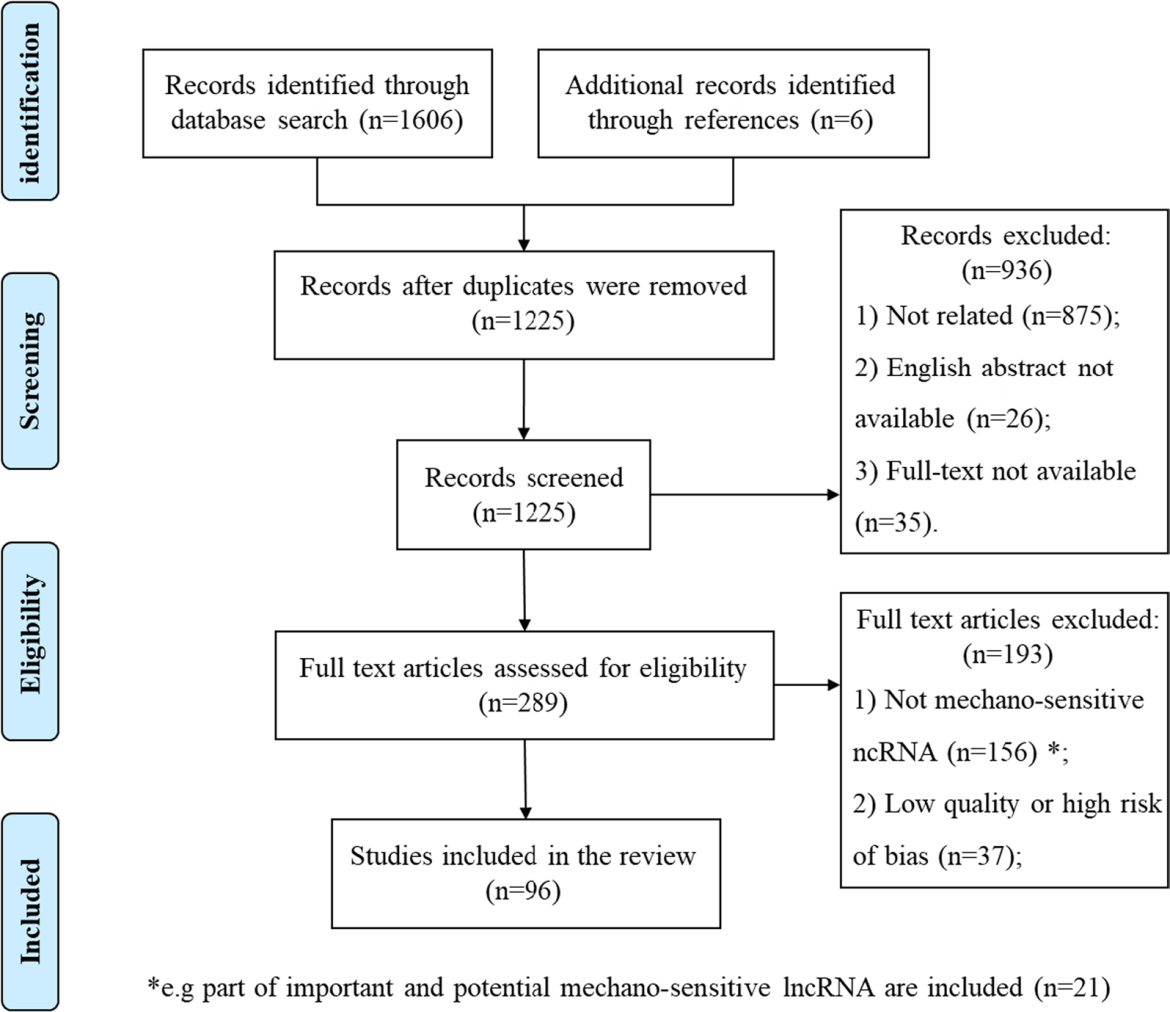
Study selection of this review

The initial literature search yielded 1,612 papers. After eliminating duplicates, 462 publications were chosen, of which 858 research were disqualified for being irrelevant. 241 of the remaining 367 articles were excluded after scrutinizing full-text due to their poor caliber or lack of applicability. This review eventually contained 126 papers (Fig. 1).

## Results

### Synthesis of results

According to the results of the entitled studies, 19 studies revealed and discussed different types of mechano-sensitive ncRNA research. We included as many other non-mechanical stimulation studies as possible in our study based on mechano-sensitive ncRNA to describe as completely as possible the existing functional studies of these mechano-sensitive ncRNA. A total of 49 studies focused on the mechanical sensitivity of the miRNA, its validated target genes, and its role in bone remodeling. Moreover, a total of 26 studies focused on the mechanical sensitivity of the lncRNA, its validated target genes, and its role in bone remodeling. Finally, the remaining 21 studies discuss lncRNA, which plays an important role in the functional conditions of OBs and OCs, but for which the mechanical sensitivity is not well characterized.

## Mechanical and biomechanics mechanisms of OTM

### Mechanical transfer of orthodontic force

**Fig. 2:**
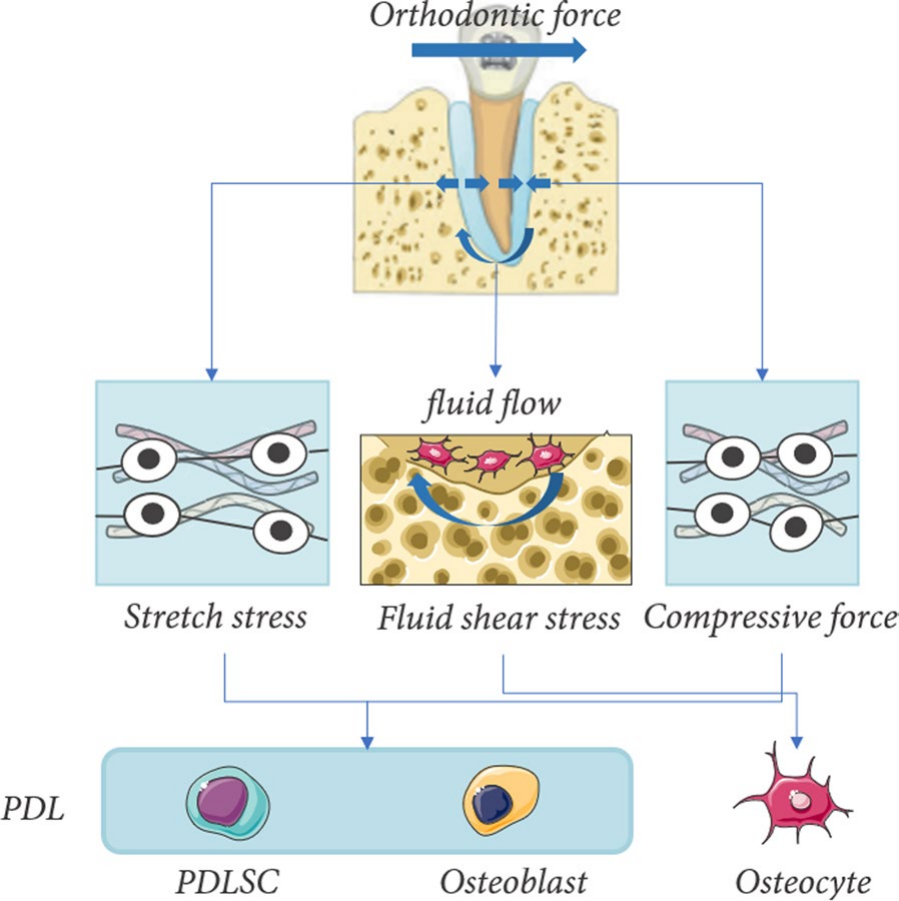
The mechanical transfer of orthodontic force and corresponding effector cells. During OTM, the side to which the orthodontic force is directed is subject to significant compressive stress, whereas the opposite side is subject to strong stretch stress. Meanwhile, as bone canaliculus are compressed and narrowed, the fluid in the bone flows from the compression side to the stretch side [16]

The prevalent theories of OTM assume that the communication network between PDLCs and osteocytes, which respond to mechanical signals as the key sensors, governs bone remodeling despite the fact that the mechanical mechanism of OTM is not fully elucidated [17]. Based on the growing evidence, we are committed that, among PDLC, PDLSC [18] and OB act as effector cells responding to stretch stress and compressive force, while the fibroblast, which makes up the bulk of PDLCs, plays the primary role of mechanical transfer. Thus, it is more appropriate to consider PDLSC, OBs, and osteocytes as the mechanical sensor cells of OTM. Orthodontic force is transmitted into strain stress, compressive force, and fluid shear stress during OTM. The three forms of force have their specific concentration areas and corresponding effector cells (Fig. 2). Constriction of the PDL microvasculature results in the initial inflammatory event at the compression site, which leads to focal necrosis with a histological appearance known as hyaline degeneration [1]. Conversely, tensive regions in orthodontic samples are frequently characterized as being predominantly osteogenic with no apparent inflammatory component [1]. Mechanical signal transduction occurs when effector cells detect MF and transform mechanical impulses into biochemical signals via an array of mechano-receptors and signaling channels.

### Mechanical signal transduction in the cell

**Fig. 3:**
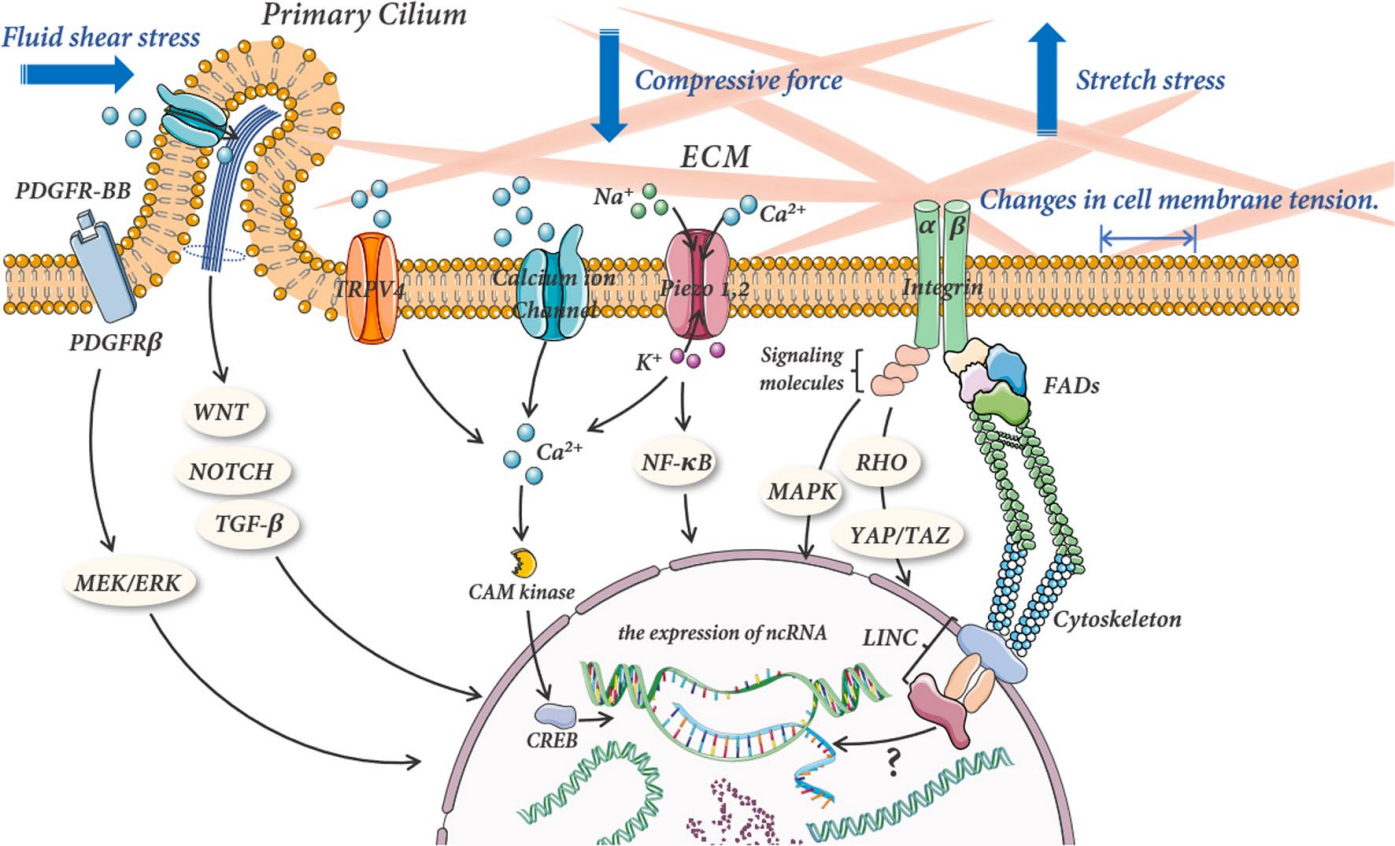
The mechanical transduction of mechanical stimulation and its effect on the expression of ncRNA. Different types of mechanical stimuli modulate cell membrane tension and further motivate calcium channels and other cation channel opening via the ECM and primary cilia. Additionally, integrins could directly sense mechanical signals through the ECM, which alter the expression of ncRNA by the activation of downstream signal pathways and mechanical transmission of the cytoskeleton

Mechanical signal transduction is the capability of cells to detect mechanical cues from their microenvironment and convert them into biochemical signals that trigger cellular responses (such as adaptive transcript) [19] (Fig. 3). On the cell surface, mechano-receptor proteins sense changes in the external mechanical environment via the phosphorylation-induced conformational changes and convert them into biochemical events within the cell. This results in changes in gene expression in the nucleus, ultimately activation or inhibition of a series of downstream signals that alter cell morphology.

The mechano-receptor can be broadly classified into two groups: proximal mechano-sensors, consisting of integrin, primary cilia, ion channels, and the cytoskeleton, and nuclear envelope proteins [19, 20]. Integrins are heterodimers composed of α and β subunits non-covalently bound to sense physical or biochemical stimuli in the ECM by binding to extracellular ligands [21, 22] and further, via conformational changes, mediate the transmission of mechanical signals to cells to induce their biological activities. Like integrins, primary cilia are ubiquitous in osteocytes, OBs, and MSCs and respond to mechanical stimulation and induction of coordinated load [20, 23, 24]. When mechanical stimulus bends the primary cilia, the increased tension on the membrane activates mechano-sensitive ion channels, resulting in the influx of Ca^2+^ into the cell, after which cell is mechanically stimulated [24]. Piezo1 and Piezo2 have been widely recognized as significant mechano-sensitive channels in bone metabolism [25–28]. However, they are not necessary mechano-channels in OBs and osteocytes. Other channels, such as TRPV [29, 30], can also direct the influx of cation under membrane tension, converting mechanical cues into biological signals. Moreover, the cytoskeleton, a highly nonlinear, intracellular network structure composed of actin fibers, microtubules, and intermediate filaments, acts as a pivotal role in sensing MF via deformation and reformation [31]. And then, the microtubule cytoskeleton and F-actin transmit mechanical signals to the nuclear envelope, which comprises the outer and inner nuclear membranes [32]. In the outer nuclear membrane, the linkages between the nesprin domains and INM SUN protein, also known as the LINC, transmit tension and shear forces across the nuclear membrane, regulating gene expression [33, 34].

When cells sense mechanical stimuli, various intracellular mechanical signaling pathways are activated, thereby altering the functions of cell proliferation, migration, and differentiation. Numerous in vitro and in vivo models have revealed the involvement of the Wnt pathway in mechanical stress, making it one of the most significant pathways in bone metabolism, and the mechanical effects of Wnt are mediated, in large part, through mechano-sensitive extracellular modulators [35]. Studies revealed that the mechanical loading could inhibit the expression of sclerostin [36, 37] and DKK1 [38, 39], the antagonists of Wnt co-receptor LRP, in osteocytes, and then trigger the upregulation of Wnt. Moreover, Ziouti et al. [40] first reported that the Notch pathway is stimulated under mechanical stress. They found that the application of cyclic stretching to hBMSCs enhanced the expression of osteogenic genes HES1 and RUNX2. Another significant mechanical signal pathway is ERK, which belongs to the family of MAPKs [41]. Liu et al. [42] demonstrated that the ERK5-AKT-Foxo3a pathway plays a crucial role in the anti-apoptotic effect in OBs via FSS-mediated caspase 3 suppression. With further studies, the RhoA signaling pathway, a crucial regulator of the actin cytoskeleton, has been recognized as the core of mechano-transduction. Stretch stimulation could activate the RhoA/ROCK) and YAP/TAZ pathway, facilitating osteogenesis and simultaneously inhibiting adipogenesis [43]. Additionally, there are other signaling pathways [44, 45] and the crosstalk [46] between various pathways in response to mechanical cues.

### Mechano-sensitive ncRNAs in mechanical regulation

With advances in cognitive recognition and cellular detective technologies, ncRNAs, including miRNAs and lncRNAs, are pivotal in regulating gene expression and translation, which is an unprecedented regulatory mechanism of the OTM process. Regarding the essence of OTM is force-induced bone remodeling, it is well understood to focus on mechano-sensitive ncRNAs when probing the regulatory ncRNAs network of OTM, as mechano-sensitivity implies a more intimate relationship with force-induced regulatory processes. For instance, via microarray assay and RT-qPCR analysis, a multitude of differently expressed MiRNAs are identified in PDLCs under MF, including miR-195-5p, -424-5p, -1297, -3607-5p, -145-5p, -4328, and -224-5p, which were core miRNAs to promote osteogenesis in PDLCs induced by tension [47]. Meanwhile, another expression profiling study revealed 9 osteogenesis-related miRNAs in stretched PDLCs, involving miR-221-3p, -138-5p, -132-3p, -218-5p, -133a-3p, -145-3p, -143-5p, -486-3p, and -21-3p [48].

The mechanism by which ncRNAs exert their regulatory functions is very complex, and current research is constantly enriching and expanding this concept. However, it can still be roughly summarized as regulating the mRNA of target genes at the transcriptional and post-transcriptional levels. More details will be discussed later (“Biogenesis, transcription, and processing of miRNAs” section and “Regulatory mechanisms of lncRNAs” section). It is broadly hopeful to clarify the regulatory mechanism of ncRNA in the near future, while how mechanical signals alter the expression of ncRNA remains to be explored. Recently, a review supposed that epigenetic modifications, especially methylation, play a prominent role in the osteogenic regulation of ncRNAs [49]. Li et al. [50] noticed that, treated with the methyltransferase inhibitor 5’-AZA, the decreased methylation levels enhanced the expression of miR-149, revealing that the epigenetic modifications could regulate the osteogenic differentiation of MSCs via miRNA.

## miRNA in bone remodeling of orthodontic tooth movement

### Biogenesis, transcription, and processing of miRNAs

MiRNAs, a family of small single-stranded ncRNAs of approximately 20–24 nucleotides [51–53], regulate gene expression via facilitating degradation or suppressing translation of target mRNAs at both transcriptional and post-transcriptional levels [54]. Silicon analysis shows that approximately 30% of protein-coding genes contain target binding sites of miRNA in their 3’UTR, and a single miRNA can generally modulate the expression of hundreds of genes [53, 55]. MiRNAs have emerged as crucial regulators in a variety of biological processes [53, 56]. Moreover, it is well known that miRNAs regulate cell differentiation, survival, and death, which is crucial for musculoskeletal activities [57].

### Mechano-sensitive miRNAs in bone remodeling of orthodontic tooth movement

MiRNAs, especially mechano-sensitive miRNAs, are frequently associated with the process of bone remodeling during OTM [52, 58]. Networks of genes that control how MSCs, OBs, and OCs differentiate and function may be impacted by changes in miRNA expression levels brought on by MF.

Here, we broadly categorize these miRNAs based on their function in bone remodeling: (1) miRNAs that regulate the function and differentiation of osteogenesis (2) miRNAs that regulate the function and differentiation of osteoclastogenesis. MiRNAs such as miR-34, -139-5p, -33-5p, -503-5p, -1246, -195-5p, -424-5p, and -145-5p are associated with osteogenesis, while microRNAs such as miR-29 and -103 are associated with OB cell strain. Particularly, miR-21 and -29 pose impacts on both osteoblastic and osteoclastic processes. The mechanical sensitivity of the miRNAs, their verified target genes, and their function in bone remodeling are all displayed in Table 1.

**Table 1:** Role of mechanical force microRNAs in bone remodeling

miRNAs	Response to MF	Direct gene targets	Pathway(s) regulated	Role in bone remodeling	References
miR-34a	hMSCs, CS↑	LDHA↓	Cellular anaerobic glycolysis↓	Inhibit OB differentiation	[59]
	hBMSCs	DKK1↓	Wnt↓	Inhibit OB differentiation	[60]
	MC3T3-E1, FSS↓	FGFR1	Runx2↓	Inhibit OB proliferation and promote apoptosis	[61]
	hMSCs	JAG1	Notch signaling↓	Inhibit OB differentiation and proliferation	[62]
miR-103	hFOB1.19, CS↓	Runx2	Runx2↓	Inhibit OB proliferation	[63]
miR-139-5p	mOB, CS↓	MACF1↓	Wnt/β-catenin or BMP↓	Inhibit OB differentiation	[64]
miR-33-5p	MC3T3-E1, CS↓ FSS↑	Hmga2↓	Extensive regulation	Attenuated the inhibition of OB differentiation induced by Hmga2	[65, 66]
miR-1246	PDLSCs, CS↑	GSK3β, Axin2	Wnt/β-catenin↑	Promote OB differentiation	[58, 67]
miR-503-5p	BMSCs, CS↓	SORBS1	Unknown	Inhibit OB differentiation and proliferation	[68, 69]
miR-494-3p	MC3T3-E1, CF↑	FGFR2、 ROCK1↓	FGF	Inhibit OB proliferation	[70]
miR-195-5p	PDLCs, CS↓	WNT3A BMPR1A FGF2	WNT, FGF, BMP↓	Inhibit osteogenic differentiation of PDLCs	[47, 71, 72]
miR-424-5p	PDLCs, CS↓	WIF1	Wnt/β-catenin↓	Inhibit osteogenic differentiation of PDLCs	[47, 73]
miR-145-5p	PDLCs, CS↓	SEMA3A	Wnt/β-catenin↓	Inhibit osteogenic differentiation of PDLCs	[47, 74]
miR-3198	PDL, CS↓CF↑	Unknown	OPG↓	Inhibit osteoclastogenesis	[75]
miR-21	mPDLSC, Orthodontic force↑	PDCD-4↓	Unknown	Attenuate the repression of PDCD-4 to OC differentiation	[76]
	PDLSC, CS↑	ACVR 2B	TGF-β pathway↑	Promote stretch-induced PDLSC osteogenesis	[77]
	hUMSCs	PI3K-AKT-GSK3β	β-catenin/RUNX-2↑	Promote osteogenesis of MSCs	[78]
	MC3T3-E1	Smad 5	BMP, TGF-β ↑	Promote the level of OB differentiation and matrix mineralization	[79]
	BMSCs	Smad 7	Smad7-Smad1/5/8-Runx2 pathway↑	Promote the bone formation of BMSCs	[80]
	PDLSC	HIF-1	VEGF	Promote PDLSC osteogenesis	[81, 82]
	mOB	FasL	Fas-FasL	Promote osteoclastogenesis and suppress osteoclastic apoptosis	[83]
miR-29 family (a,b,c)	PDLCs, CS↓ CF↑	COL1A1 COL3A1 COL5A1↓	Unknown	Unknown	[84]
	OC	Calcr↓	Calcr-cAMP-Ctsk↑	Promote bone resorption	[85]
	RAW264.7	Cdc42, Srgap2, Gpr85 Cd93 Nfia	Extensive regulation	Promote the differentiation and function of OC	[86]

*miR* microRNA, *CS* cyclic stretch stress, *CF* compressive force, *FSS* fluid shear stress, *hMSC* human mesenchymal stem cell, *LDHA* lactate dehydrogenase-A, *OB* osteoblast, *hBMSC* human bone marrow mesenchymal stem cell, *DKK1* Dickkopf-1, *MC3T3-E1* Mouse osteoblastic cell line, *FGFR1* Fibroblast Growth Factor Receptor 1, *Runx2* Runt-related transcription factor 2, *JAG1* jagged canonical Notch ligand 1, *hFOB1.19* human osteoblastic cell line, *mOB* mouse osteoblast, *MACF1* microtubule actin crosslinking factor 1, *Hmga2* high mobility group AT-hook 2, *PDLSC* human periodontal ligament stem cell, *GSK3β* glycogen synthase kinase 3 beta, *Axin2* axis inhibition protein 2, *PDLC* human periodontal ligament cell, *SORBS1* Sorbin and SH3 domain-containing protein 1, *FGFR2* Fibroblast Growth Factor Receptor 2, *Rock1* Rho-associated coiled-coil containing protein kinase 1, *WNT3A* WNT family member 3A, *BMPR1A* the bone morphogenetic protein receptor-type IA, *SEMA3A* semaphorin 3A, *mPDLSC* mouse periodontal ligament cell, *PDCD-4* programmed cell death-4, *hUMSCs* Human umbilical cord-derived mesenchymal stem cells, *PI3K* Phosphoinositide 3-kinase, *AKT: GSK3β* glycogen synthase kinase 3 beta, *Calcr* calcitonin receptor, *Cdc42* cell division cycle 42, *Srgap2* SLIT-ROBO Rho GTPase-activating protein, *Gpr85* G protein-coupled receptor 85, *Cd93* complement component 1q receptor 1, *Nfia* nuclear factor I/A, *OC* osteoclast

### miRNAs regulate the function and differentiation of osteogenesis

**Fig. 4:**
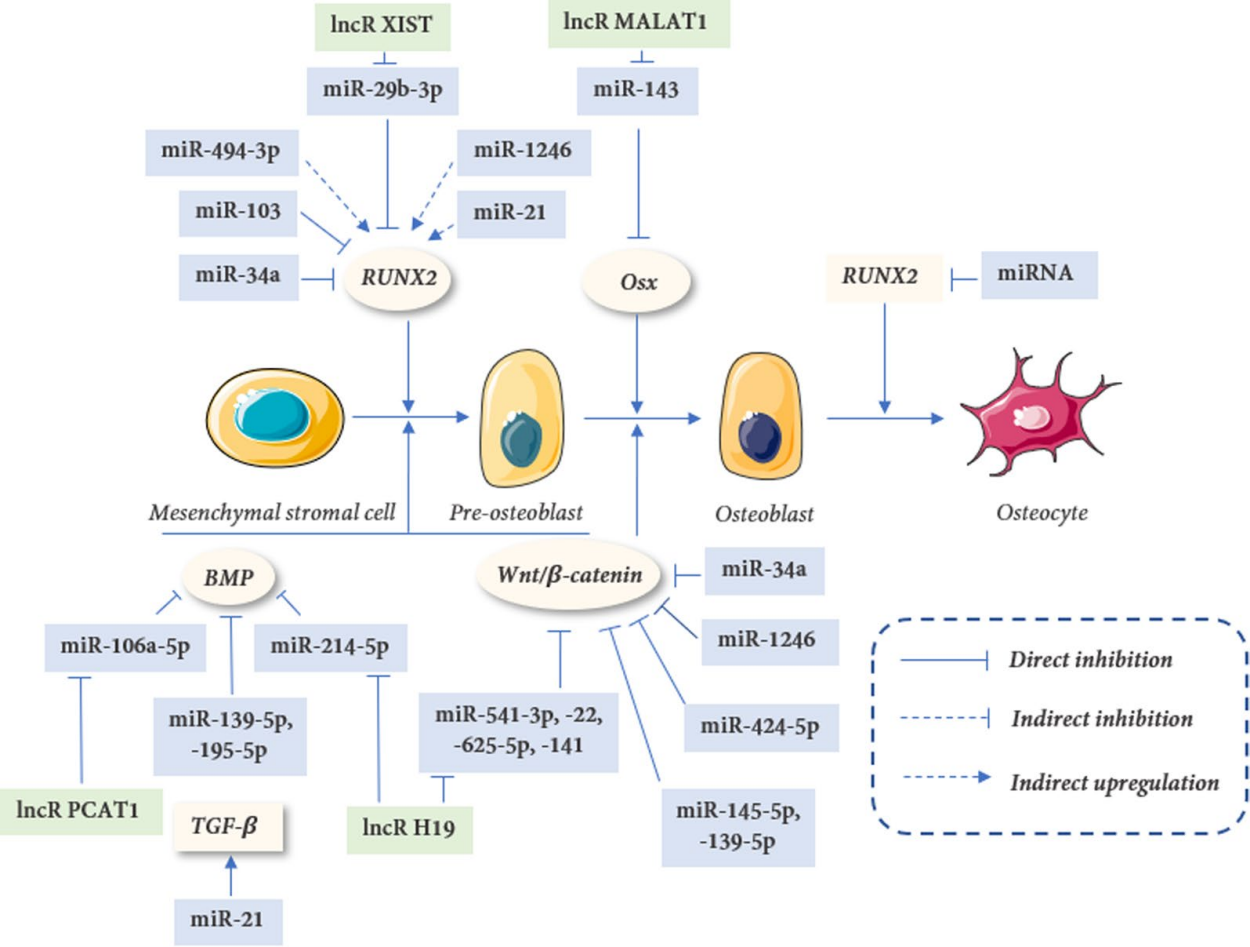
Mechano-sensitive miRNAs and lncRNAs in osteogenesis: The microRNAs and long non-coding RNAs are regulated by mechanical force and are implicated in osteogenesis through regulating gene targets in mesenchymal stem cells, pre-osteoblasts, osteoblast cells, and osteocytes

These miRNAs are facilitating or suppressing the differentiation of MSCs, PDLSCs, and the function of OBs (Table 1 and Fig. 4).

#### miR-34a

MF induces upregulated expression of MiR-34a in vivo and in vitro, which may be associated with GSK3β [87]**.** Wenwen Yu et al. [87] supplied miR-34a via N-acetyl-L-leucine-modified polyethylenimine exhibiting satisfactory biocompatibility and great miRNA transfection efficacy in rat BMSCs and alveolar bone, which render an excellent delivery system of ncRNA. Zeng et al. [60] proved that miR-34a regulated the Wnt signaling pathway via directly targeting DKK1. Overexpression of miR-34a inhibited late OB differentiation of hMSCs in vitro, which demonstrated the roles of miR-34a in the regulation of OB differentiation [59]. Mechanically, miR-34a inhibits OB differentiation by targeting 3'UTR of LDHA. Additionally, the up‐regulation of miR‐34a attenuated FSS‐induced promotion of OB proliferation and survival via FGFR1 [61]. Chen et al. [62] identified miR-34a exhibited unique dual regulatory effects on both hMSC proliferation and OB differentiation. They uncovered that miR-34a down-regulated Notch signaling to inhibit OB differentiation by targeting JAG1, while suppressing cell proliferation by targeting several cell cycle genes, which cause a halt in the G1 and G2 phases of the cell cycle. Conversely, down-regulated miR-34 expression was positively correlated with the expression of MMP (MMP-2, MMP-9, and MMP-14), which promote the degradation of ECM and are thus essential for bone formation [88].

#### miR-103a

Bioinformatic analyses, followed by qRT-PCR validation, revealed that miR-103a is distinctly down-regulated under cyclic mechanical stretch [63]. Mutation of putative miR-103a binding sites in Runx2 mRNA abolishes miR-103a-mediated repression of the Runx2 3'UTR via luciferase reporter activity, revealing that miR-103a regulates OB differentiation by directly targeting Runx2 [63]. Additionally, they discovered the inhibitory role of miR-103a in controlling osteogenesis in hindlimb unloading mice, as pretreatment with antagonist miR-103a partially reversed the osteoporosis brought on by mechanical unloading [63]. Inhibiting miR-103a therapeutically may be an effective anabolic tactic for treating skeletal diseases brought on by pathological mechanical loads.

#### miR-139-5p

Chen et al. [64] found that, in primary OBs, the miR-138-5p level was continually declined in response to cyclic stretch MF, accompanied by enhanced capability of OB differentiation. The results validated miR-138-5p as a mechano-responsive miRNA that is negatively correlated with OB differentiation in vitro. Dual-luciferase reporter assay and further in vitro confirmation suggest that MACF1, positively regulating OB differentiation by activating the β-catenin/TCF1-Runx2 axis [89], is a key target of miR-138-5p for detecting different mechanical stimuli and mediating OB differentiation.

#### miR-33

Mechanical stretch decreased miR-33 in MC3T3-E1 cells, which indicating it as one of the mechano-responsive miRNAs [65]. Conversely, miR-33-5p, one subtype of miR-33, was significantly upregulated under FSS, and the knockdown of miR-33-5p partially prevented FSS-induced OB differentiation [66]. Given that miR-33-5p positively regulates osteoblast differentiation, Wang et al. [66] investigated the roles of miR-33-5p in OB activity under MF. The mechanical unloading-induced suppression of MC3T3-E1 osteogenic differentiation was somewhat mitigated by overexpressing miR-33-5p, and Hmga2, the miR-33-5p target, was shown to adversely influence OB development.

#### miR-1246

MiR-1246 was the miRNA with the highest expression level among those miRNAs that were shown to be elevated in stretched PDLSCs [58]. Peng et al. [67] identified that miR-1246 suppresses the production of GSK3β and Axin2, which results in the activation of the Wnt/β-catenin pathway and inflammation in condylar chondrocytes.

#### miR-503-5p

Liu et al. [68] reported that miR-503-5p functions as a mechano-sensitive miRNA and inhibits the osteogenic differentiation and bone formation of BMSCs while subjected to mechanical stretch. Furthermore, SORBS1 was detected as a target of miR-503-5p, which was highly increased in osteogenesis [69]. A prior study showed that CAP protein encoded by the SORBS1 gene, combined with vinculin in the integrin complex and mediates homeostatic adaptation in response to external forces [90, 91]. Numerous reports have confirmed that the knockdown of SORBS1 suppressed the osteogenic differentiation process of stem cells [92, 93].

#### miR-195-5p, miR-424-5p, miR-145-5p, and miR-224-5p

Chang et al. [47] found that miR-195-5p, -424-5p, -1297, -3607-5p, -145-5p, -4328, and -224-5p were core miRNAs involved in tension-induced bone formation. Under cyclic stress strain (CTS), miR-195-5p overexpression greatly reduced the ability of PDLCs to differentiate, whereas miR-195-5p functional inhibition had the opposite impact. Additional research shown that miR-195-5p directly targets the proteins WNT3A, FGFR2, and BMPR1A, which are crucial for osteogenic activity and stability [71]. Early expression of miR-195-5p inhibits OB differentiation and mineralization by targeting BMP signaling [72]. Wei and his colleagues [73] revealed the evidence for miR-424-5p as a novel reagent for the treatment of osteoporosis by demonstrating that exosomes overexpressing miR-424-5p attenuated osteogenic development via WIF1/Wnt/-catenin in vivo. Data indicate that miR-145 may play a critical role in the osteogenic differentiation of MSCs because it may adversely affect SEMA3A, a positive regulator of osteogenesis. [74]. Besides, it was found that upregulated miR-224-5p enhanced Pai-1 expression to impair OB differentiation [94].

#### miR-21

As one of the most extensively researched miRNAs, miR-21 has drawn interest from researchers in a variety of domains [95], including development, cancer, stem cell biology, and aging, ever since it was shown to be the miRNA most often and robustly expressed in a variety of human tissues. MiR-21 has been shown in several investigations to regulate osteogenic differentiation in response to mechanical stimulation [51, 52, 77] and previously reported to mediate stretch-induced osteogenic differentiation of hBMSCs and PDLSCs [77] and support OCs differentiation in vitro [58]. According to bioinformatics prediction results, the target genes of miR-21 are considerably enriched in Jak-STAT and MAPK signaling pathways, which are connected to osteogenic differentiation [58]. Wei et al. [77, 96] confirmed that mechano-sensitive miR-21 can promote stretch-induced PDLSC osteogenesis by acting on the transmembrane serine/threonine receptor kinase ACVR2B, which is an essential component of the TGF pathway and affects cell growth and differentiation in a variety of biological functions [97]. Meng and colleagues [78] demonstrated that miR-21 and its target PI3K-AKT-GSK3β pathway were crucial for the osteogenic differentiation of MSCs by maintaining β-catenin. MiR-21 enhances the activity of the PI3K-AKT signaling pathway, which facilitated the phosphorylation of GSK3β to stabilize β-catenin in the cytoplasm to activate RUNX-2 mediated osteogenesis of MSCs. In MC3T3-E1 cells, miR-21 has also been shown to support osteogenic differentiation and matrix mineralization by directly suppressing Smad7 [79]. Interestingly, forced expression of miR-21 regulates the levels of Smad 7 by inhibiting translation, rather than by accelerating mRNA decay [96]., Zhang et al. [81] discovered that miR-21 upregulated HIF-1 expression in PDLSCs and encouraged osteogenic differentiation, while miR-21 inhibitors down-regulated HIF-1 expression and osteogenic markers. The results of these studies revealed that miR-21 might have a diametric role in osteogenesis, depending on the cell types and manner of mechanical stimuli.

#### miR-29

CTS decreased the expression of miR-29 family members, while compression force increased miR-29 family expression in PDLCs [84]. Additionally, transient transfection of a miR-29b mimic decreased the expression of Col1a1, Col3a1, and Col5a1 in mouse PDLCs, whereas transfection of a miR-29b inhibitor increased the expression of these genes, indicating that miR-29b is involved in the control of ECM formation [84].

### miRNAs regulate the function and differentiation of osteoclastogenesis

**Fig. 5:**
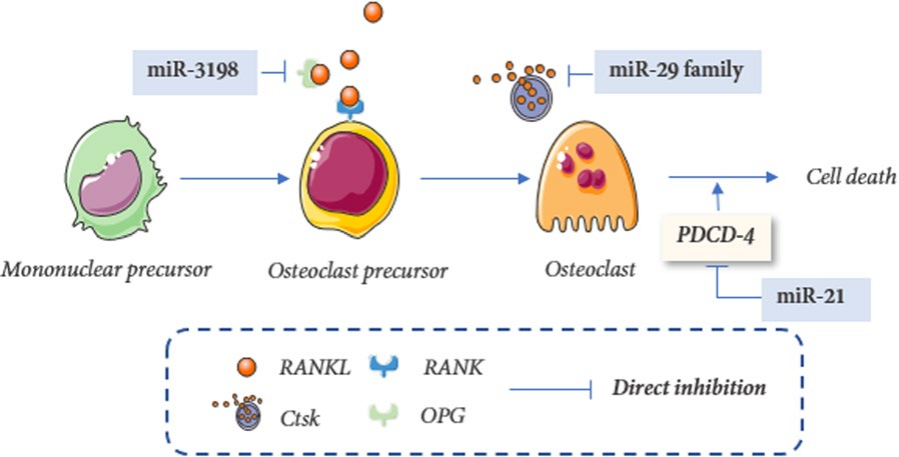
Mechano-sensitive miRNAs in osteoclastogenesis: The microRNAs are regulated by mechanical force and are implicated in osteoclastogenesis through regulating gene targets in mononuclear precursors, osteoclast precursors, and osteoclast cells

A cluster of miRNAs is associated with osteoclastic differentiation and bone resorption (Table 1 and Fig. 5).

#### miR-3198

Real-time RT-PCR confirmed that miR-3198 decreased under compression and increased under tension [75]. Further experiments were observed that miR-3198 mimics reduced OPG expression at the tensive sides, whereas the miR-3198 inhibitor rescued the compression-mediated down-regulation of OPG, revealing that miR-3198 negatively regulated OPG expression in response to mechanical stress [75]. However, neither the mimic nor the miR-3198 inhibitor had an impact on RANKL expression [75]. According to an investigation of gingival crevicular fluid, miR-3198, particularly when combined with miR-4299, exhibited a notable sensitivity and specificity for evaluating periodontitis [98].

#### miR-21

MiR-21 deficiency blocked not only bone formation but also bone resorption under physiologic conditions. The RANKL-induced differentiation of OCs from bone marrow monocytes is mediated by miR-21. C-Fos and PU.1, which are transcription factors for osteoclastogenesis, bind to the AP-1 and PU.1 binding sites in the miR-21 promoter to trigger miR-21 transcription [99]. miR-21 is a critical prerequisite for down-regulation of PDCD-4, one of the repressor proteins of osteoclastogenesis [100], via binding PDCD-4-3'-UTR [101], which facilitates the translation of transcription factors and osteoclast-specific markers of osteoclastogenesis. Previous studies have reported that many factors, including smad5, can influence osteoclast OC differentiation when mechanical force MF is applied [102]. Li et al. [80] reported that miR-21 could boost the expression of smad5, which down-regulates the expression of OC serum markers, suggesting that smad5 may be one of the targets of miR-21 to affect bone resorption. Further research indicated miR-21 promotes the bone formation of BMSCs and this process is regulated, in part, by the Smad7-Smad1/5/8-Runx2 pathway [80]. Nakamura et al. [83] confirmed that FasL was a target of miR-21 and estrogen down-regulated miR-21 biogenesis so that protein levels of FasL are elevated post-transcriptionally, thus inhibiting osteoclastogenesis and inducing osteoclastic apoptosis. In conclusion, the results revealed that miR-21 played significant roles both in osteogenic differentiation and OC differentiation during OTM [103, 104].

#### miR-29

Shin et al. reported that, in RANKL-driven osteoclastogenesis, miR-29-3p promotes resorption by targeting calcitonin receptor (Calcr) which negatively regulated bone resorption. Consequently, inhibition of miR-29-3p in the myeloid lineage increases response to calcitonin and suppresses the OC activity. [85]

By means of luciferase 3'-UTR reporter assays and specific miR-29 inhibitors, research fellows [86] demonstrated that miR-29 negatively regulates mRNAs critical for the cytoskeletal organization, including Cdc42 [105] and Srgap2. Moreover, miR-29 targets mRNAs associated with the macrophage lineage: Gpr85, Nfia, and Cd93 [86]. Moreover, it was observed that overexpressing miR-29b improved the capacity of BMSCs to proliferate and migrate in rats with castration-induced osteoporosis [106]. This improvement may be connected to the activation of the PI3K/AKT and TGF-/Smad signaling pathways, indicating that miR-29 performs a variety of regulatory functions in a variety of settings.

### Brief summary

The miR-21 and miR-29 families are examples of mechano-sensitive miRNAs that have been linked to both osteoblastic and osteoclastic responses. This may be due to the fact that a single miRNA can target a large number of target mRNAs, some of which promote pro-atherogenic responses in a particular cell type or organ, while others function in a contrary way in multiple cell types, tissues, or contexts. As a result, cellular context, cell type, and environment are likely to have an impact on the overall response of miRs throughout the entire body in vivo.

## LncRNAs in bone remodeling

The lncRNAs are a group of ncRNAs with a length of more than 200 nucleotides that play an indispensable role in the regulation of gene expression at the post-transcriptional and competitively bind to specific miRNA sites to regulate their expression levels [107, 108]. Here, we discuss the mechano-sensitive lncRNA XIST, H19, and MALAT1 along with other lncRNAs studied in the context of osteogenesis and osteoclastogenesis, such as DANCR, TUG1, PCAT1, SNHG1, NEAT1, and MIRG, all of which are summarized in Table 2.

**Table 2:** A summary of lncRNAs and their physiological effects on bone remodeling

LncRNA	Major targets	Indirect targets and signaling pathway	Physiological effects	References
XIST	miR-214-3p	Unknown	Inhibit osteogenic differentiation of PDLSCs	[109]
	miR-29b-3p	RUNX2	Inhibit OB differentiation	[110]
	miR-135	RANKL	Inhibit OB differentiation	[111]
	miR-375-3p	AKT/mTOR	Inhibit OB differentiation	[112]
DANCR	Unknown	WNT	Promote osteogenic differentiation of PDLSC	[113]
	Unknown	EZH2↓, Runx2↑	Increase OB proliferation and decrease OB apoptosis	[114]
	miR-758	Notch2	Suppress osteogenic differentiation of PDLSC	[115]
	Jagged1	Notch	Facilitate osteoclast formation and root resorption	[116]
TUG1	miR-222-3p	Smad2/7	Facilitate osteogenic differentiation of PDLSC	[117]
	Unknown	Wnt/β-catenin	Promote OB proliferation and differentiation	[118]
	miR-545-3p	CNR2	Promote OB proliferation and differentiation	[119]
	miR-34a	FGFR1	Promote OB proliferation and inhibit apoptosis	[61]
	Unknown	MafB	Stimulate osteoclast formation	[120]
PCAT1	miR-106a-5p	BMP2	Promote osteogenic differentiation of PDLSC	[121]
H19	miR-675	TGF-β1/Smad3/HDAC	Promote osteogenic differentiation of hMSC	[122]
	miR-22, -141	Wnt/β-catenin	Potentiate osteogenesis	[123]
	miR-138	FAK	Unknown	[124]
	miR-188	LCoR	Promote osteogenic differentiation of mBMSC	[125]
	miR-19b-3p	Unknown	Promote cell proliferation and osteogenic differentiation of BMSCs	[126]
	miR-675	APC	Promote osteogenic differentiation of BMSC	[127]
	miR-149	SDF-1	Stimulates osteogenic differentiation of BMSC	[128]
	miR-185-5p	IGF1	Promote matrix mineralization	[129]
	miR-140-5p	SATB2	Promote osteogenic differentiation of BMSC	[130]
	miR-467	HoxA10	Promote osteogenic differentiation of BMSC	[131]
	miR-106	Angpt1	Promote osteogenesis and angiogenesis	[132]
	miR-625-5p	Wnt/β-catenin	Promote BMSC proliferation and osteogenic differentiation	[133]
	miR-541-3p	Wnt/β-catenin	Promote osteogenic differentiation of BMSC	[134]
	miR-532-3p	SIRT1	Promote osteogenic differentiation of BMSC	[135]
	miR-214-5p	BMP2	Promote osteogenic differentiation of BMSC	[136]
MALAT1	miR-143	Osterix	Promote osteogenic differentiation of BMSC	[137]
	miR-155-5p	ETS1	Promote osteogenic differentiation of PDLSC	[138]
SNHG1	miR-101	DKK1	Suppress osteoblast differentiation	[139]
	miR-181c-5p	SFRP1/Wnt/β-catenin	Inhibit OB differentiation and angiogenesis and promote OC formation	[140]
	KLF2	Unknown	Inhibit osteogenic differentiation of PDLSC	[141]
NEAT1	miR-7	PTK2	Stimulate osteoclastogenesis	[142]
MIRG	miR-1897	Unknown	Stimulate osteoclastogenesis and bone resorption	[143]

*lncRNAs* long non-coding RNAs, *XIST* X-inactive specific transcript, *DANCR* Differentiation antagonizing non-protein coding RNA, *EZH2* Enhancer of zeste homolog 2, *TUG1* Taurine upregulated 1, *CNR2* cannabinoid receptor 2, *MafB* musculoaponeurotic fibrosarcoma oncogene homolog B, *PCAT1* prostate cancer-associated ncRNA transcript 1, *FAK* Focal adhesion kinase, *LCoR* Ligand-dependent corepressor, *SDF-1* Stromal Cell Derived Factor 1, *IGF1* insulin-like growth factor 1, *SATB2* Special AT-rich sequence-binding protein 2, *HoxA10* homeobox A10, *Angpt1* angiopoietin 1, *SIRT1* sirtuin 1, *MALAT1* metastasis-associated lung adenocarcinoma transcript 1, *ETS1* E26 transformation specific-1, *SNHG1* Small nucleolar RNA host gene 1, *DKK1* Dickkopf-1, *SFRP1* secreted frizzled-related protein 1, *KLF2* Kruppel-like factor 2, *NEAT1* nuclear paraspeckle assembly transcript 1

### Regulatory mechanisms of lncRNAs

The regulatory mechanisms of lncRNAs are quite sophisticated. Four levels, epigenetic, transcriptional, post-transcriptional, and other regulatory, can be used to categorize the action mechanisms of lncRNAs. Three general functional roles for lncRNAs in bone remodeling are as follows: They (1) modulate signaling pathways to control bone remodeling; (2) act as miRNA sponges or precursor structures to control bone remodeling; and (3) facilitate epigenetic modification to control bone remodeling.

### LncRNA in the regulation of the bone formation

#### LncRNA XIST

A novel lncRNA X-inactive specific transcript (XIST) has been recently reported to regulate the development of many diseases [144], such as colorectal cancer [145] and non-small cell lung cancer [146]. Besides, lncRNA XIST is sensitive to strain stress in PDLSCs [147]. Feng et al. [109] revealed that inhibiting XIST lowered the protein expressions of OB markers ALP, OCN, and RUNX2, as well as ALP activity, thus hypothesizing overexpression of XIST promotes osteogenic differentiation. Further luciferase reporter, RNA immunoprecipitation, and RNA pull-down assays demonstrated that lncRNA XIST enhances osteogenic differentiation of PDLSCs via sponging miRNA-214-3p [109]. Particularly noteworthy was the finding that osteoporosis patients’ plasma and monocytes had much higher levels of XIST than did healthy controls. [148]. Yu et al. [110] reported that XIST negatively regulates nicotinamide N-methyltransferase by sponging miR-29b-3p to restrict the osteogenic differentiation of BMSCs. Knockdown of XIST induces the differentiation of osteoblast-like cells via regulation of the miR-135/CREB1/TNF-α/RANKL [111] and miR-375-3p/AKT/mTOR [112] axis, representing an attractive therapeutic strategy to accelerate bone remodeling.

#### LncRNA DANCR

Anti-differentiation noncoding RNA was discovered as a progenitor differentiation-associated lncRNA by Kretz and colleagues [149]. However, since ANCR has been named Angelman syndrome chromosome region, it is recommended to modify anti-differentiation noncoding RNA to differentiation antagonizing non-protein coding RNA (DANCR). Therefore, both ANCR and DANCR refer to the same gene in the early studies. To avoid misunderstanding, we will use DANCR as the name of this gene throughout the rest of this article. In 2018, Jia et al. [113] showed that the down-regulation of DANCR fosters osteogenic differentiation of PDLSCs via the WNT signaling pathway. Study [114] using transfection of siRNA-DANCR (si-DANCR) in OBs isolated from mice models of postmenopausal osteoporosis showed that si-DANCR significantly promoted the proliferation, ALP activity, calcium deposition of OBs, and decreased apoptosis via down-regulated EZH2 and upregulated RUNX2 and Osterix. Peng et al. [115] further showed that DANCR regulated osteogenic differentiation of PDLSCs via the miR-758/Notch2 axis.

#### LncRNA TUG1

Taurine upregulated 1 (TUG1), a 7.1-kb lncRNA located at chr22q12.2, was a lncRNA that was reported first in a study exploring the differential expression of lncRNAs in DNA damage-induced cell death in Hela cells [150]. Through the inactivation of the Wnt/-catenin pathway, Yang et al. [118] recently demonstrated that TUG1 knockdown reduced OB proliferation and differentiation. Additionally, TUG1 accelerated the proliferation and differentiation of osteogenic precursor cells (hFOB1.19 cells) by sponging miR-545-3p, which directly targets CNR2 [119]. In the osteogenic differentiation of PDLSCs, Wu [117] revealed that TUG1 accelerates the osteogenic differentiation by sponging miRNA-222-3p to regulate Smad2/7. Further, Wang et al. [61] validated that lncRNA TUG1 promoted OB proliferation and inhibit apoptosis via the lncRNA TUG1/miR-34a/FGFR1 axis, in which TUG1 acted as a ceRNA to interact with miR-34a and upregulate FGFR1 protein expression.

#### LncRNA PCAT1

The role of prostate cancer-associated ncRNA transcript 1 (PCAT1) has been thoroughly discussed in several kinds of cancers [151, 152]. Recent findings revealed a role of PCAT1 in osteogenic differentiation of ADSCs [153]. Jia et al. [121] further investigated its potential mechanism in osteogenic differentiation of PDLSCs. Via profiling lncRNA expression analysis, they found that PCAT1 boosted ALP activity and hydroxyapatite crystal formation. What’s more, PCAT1 could upregulate its target gene BMP2 via sponging miR-106a-5p, resulting in positive regulation of osteogenic differentiation.

#### LncRNA H19

The lncRNA H19, one of the most well-known imprinted genes, is located on human chromosome 11. The role of H19 in genomic imprinting has been examined in a wide range of research during the past twenty years. Recent research studies have reported that H19 was upregulated in the strained hBMSCs and highlighted H19 as a potential modulator in OB differentiation [124].

Huang and colleagues [122] demonstrated that miR-675, which is encoded by exon 1 of H19, increased OB development of hMSCs, which in part accounted for the pro-osteogenic activity of H19, and overexpression of H19 promoted osteogenic differentiation of hMSCs in vitro. The new pathway H19/miR-675/TGF-1/Smad3/HDAC is proven by the authors to govern osteogenic differentiation of hMSCs and may be a viable target for improving bone formation in vivo after investigating the underlying mechanism.

Liang et al. [123] demonstrated that H19 functioned as a miRNA sponge for miR-141 and miR-22, which were antagonistic regulators of the Wnt/β-catenin pathway and osteogenesis. Further research found that H19 interfered with the two miRNAs’ abilities to operate, which led to the de-repression of the β-catenin gene and ultimately the activation of the Wnt/β-catenin pathway, which potentiated osteogenesis. In addition, Wu et al. [124] observed that mechanical strain may raise H19 expression, and studies have demonstrated that H19 governs hBMSCs’ tension-induced osteogenesis by functioning as a ceRNA for miR-138, which in turn upregulates the downstream gene FAK. Wang et al. [125] examine the expression of lncRNA H19, miR-188, and LCoR in mBMSCs and verified the regulatory mechanism of H19/miR-188/LCoR in osteogenic and adipogenic differentiation of mBMSCs. Luciferase reporter assay validated miR-188 directly regulated LCoR and, in vitro, overexpression of miR-188 and knockdown of LCoR inhibited osteogenic differentiation and induced adipogenic differentiation in mBMSCs. By sponging miR-188, H19 functions as a mediator of LCoR to control the balance between osteogenic and adipogenic development of mBMSCs.

A cluster of papers had revealed that H19 was involved in miR-19b-3p [126], miR-675/APC axis [127], micR-149/SDF-1 axis [128], miR-185-5p/IGF1 axis [129], miRNA-140-5p/SATB2 axis [130], miR-467/HoxA10 [131], miR-106/Angpt1 axis [132], miR-625-5p/Wnt/β-catenin axis [133], miR-541-3p/Wnt/β-catenin axis [134], miR-532-3p/SIRT1 axis [135], and miR-214-5p/BMP2 axis [136]. Additionally, the effects of the H19 on osteogenic differentiation were summarized in Zhou’s [154] and Wang’s [155] review.

#### LncRNA MALAT1

Metastasis-associated lung adenocarcinoma transcript 1 (MALAT1) is often regarded as a tumor-related lncRNA, and recent papers have reported its potential function in MSC differentiation. Noticing the down-expression of MALAT1 and upregulation of miR-143 in hBMSCs from osteoporosis patients, Gao et al. [137] found that MALAT1 positively regulated Osterix expression by sponging miR-143 to promote osteogenic differentiation of hBMSC. Another study [138] revealed that MALAT1 modulated differentiation of hPDLSCs, via acting as a ceRNA for miR-155-5p and increasing miR-155-5p targeted E26 transformation specific-1 (ETS1).

#### LncRNA SNHG1

Xiang et al. [139] revealed the role of the lncRNA small nucleolar RNA host gene 1 (SNHG1) in osteogenesis. They discovered that whereas DKK1 and SNHG1 are down-regulated during osteogenesis, miR-101 and genes associated with osteogenesis are increased, suggesting that miR-101 overexpression might lower the expression levels of DKK1 and lncRNA SNHG1. The authors confirmed that SNHG1 could suppress OB differentiation by acting as a sponge for miR-101 and by inducing upregulation of DKK1 expression levels based on prior studies about miR-101 and DKK1 functions in osteogenesis, eventually reporting a novel SNHG1/miR-101/DKK1 axis in the osteogenic differentiation process. Another paper [141] proved that SNHG1 inhibits osteoblastic differentiation via epigenetic silencing of KLF2, exploring a brand-new effect of lncRNA SNHG1 in the osteogenic differentiation of PDLSCs.

### LncRNA in the regulation of bone resorption

#### LncRNA TUG1

Du and colleagues identified the influence of TUG1 on OC differentiation [120], and experiments in vitro revealed that TUG1 stimulated OC formation by facilitating the degradation of MAFB.

#### LncRNA SNHG1

Jiang et al. [140] demonstrated the regulatory mechanism by which SNHG1 suppresses BMSC osteogenic differentiation by negatively regulating the p38 MAPK signal pathway through ubiquitination mediated by Nedd4. Additionally, Yu et al. [156] discovered that SNHG1 altered the SFRP1/Wnt/β-catenin signaling pathway by sponging miR-181c-5p, which in turn inhibited OB development and angiogenesis while concurrently boosting the production of osteoclasts.

#### LncRNA DANCR

Zhang et al. [116] investigated the effect and the expression of DANCR, miR-34a-5p, and Jagged1 on the osteoclastogenesis and root resorption of PDL cells treated with compressive force in a rat OTM model. The expression of DANCR and Jagged1 protein was significantly upregulated [116], indicating DANCR as a mechano-sensitive ncRNA. The expression of DANCR and miR-34a-5p in HEK293 cells is negatively associated, according to bioinformatics and a luciferase reporter test. Moreover, knockdown of DANCR could change the promotion effect of compressive force on osteoclastogenesis and bone resorption in vitro and in vivo experiments, while overexpression of Jagged1 reversed the effect of DANCR siRNA. In conclusion, DANCR/Jagged1 axis was a potential pathway to modulate CF-induced OC formation and root resorption.

#### LncRNA NEAT1

The expression levels of the lncRNA nuclear paraspeckle assembly transcript 1 (NEAT1) were shown to be dysregulated between osteoporotic patients and normal individuals recently; however, its molecular mechanism in bone remodeling remains unknown. To address this issue, Zhang et al. [142] studied NEAT1’s impact on osteoclastogenesis and deconstructed its underlying regulatory mechanisms, discovering that NEAT1 interacted with miR-7, interfering with miR-7-mediated regulation of PTK2.

#### LncRNA MIRG

Ling et al. [143] investigated how bone marrow macrophages (BMMs) obtained from mice femurs differentiated into OCs. Following qPCR analysis, it was discovered that BMMs undergoing osteoclastogenic induction dramatically increased the expression of MIRG and NFATC1. NFATC1, TRAP, c-FOS, CTSK, MMP-9, and DC-STAMP are some of the marker genes that are down-regulated as a result of MIRG knockdown by shRNA. These genes are also implicated in osteoclastic differentiation and bone resorption. It is confirmed that miR-1897 is one of MIRG’s targets and is negatively regulated by MIRG using bioinformatics prediction techniques. When combined, MIRG acts as a molecular sponge against miR-1897, promoting osteoclastogenesis and bone resorption.

### Brief summary

Similar to that discussed in 2.5, lncRNA DANCR, TUG1, and SNHG1 have been implicated in both osteoblastic and osteoclastic responses. Interestingly, TUG1 has contradictory roles in OBs and OCs, whereas lncRNA DANCR and SNHG1 have an obvious osteogenic or osteoclastic propensity.

TUG1 has a wide range of regulatory functions and is widely recognized in the tumor field to promote proliferation, migration, cell cycle changes and inhibit apoptosis [157], whereas upregulation of TUG1 in myocardial ischemia/reperfusion (MI/R) injury is thought to promote apoptosis [158, 159]. Based on the extensive and contradictory regulatory functions of TUG1, we speculate that TUG1 may be a relatively upstream regulator that can act on a variety of functional inhibitory signaling pathway regulators and miRNAs to play a regulatory role similar to cell activation. Cells of different kinds or in different environments have different types and expression levels of signaling pathway regulators and miRNAs, so the upregulation of TUG1 has different effects. For example, osteoblast-inhibitory miR-545-3p, miR-222-3p, and miR-34 were relatively highly expressed in osteoblastic cell lines, while the osteoclast-inhibitory regulator MafB in osteoclast cell lines had relatively high expression. Because of its extensive binding and regulatory functions, TUG1 acts on the aforementioned miRNAs and regulatory factors, resulting in contradictory effects of promoting OBs and OCs. However, the current study is not clear about the environmental factors and molecular biological mechanisms of lncRNA TUG1 expression changes, and further research is needed to clarify.

## Future perspectives

### Outstanding questions

Various in vivo mechanical stimuli are sensed by cells and transduced to initiate downstream signaling. Emerging evidence unveiled that the biomechanics of the cytoskeleton and the ECM intensively modulate gene expression patterns. But the mechanisms underlying how mechanical signals accurately affect the expression of ncRNA in the cell nucleus need to be addressed. As mentioned above, the complexity and diversity of in vivo mechanical cues present distinct patterns of flow shear stress, tensile stretch, or compressive force with diverse parameters in magnitude, duration, or frequency. Thus, it is required to elucidate, from the viewpoint of mechanobiology, how a different MF leads to the differential expression of ncRNA, and whether the difference in cell function is determined by the differential ncRNA expression.

The miRNA and lncRNA are emerging as key regulators of transcription and gene expression in diverse physiological contexts responding to the mechanical stimuli. Although we have, respectively, discussed several possible molecular pathways of miRNA and lncRNA, it is crucial to remember that they are intricately interwoven and not separate. MiR-34a and TUG1, for example, form the TUG1/miR-34a/FGFR1 axis, in which TUG1 acted as a ceRNA to interact with miR-34a and upregulate FGFR1 protein expression. These, in turn, modulate the expression of FGFR1 protein and other regulators, creating a feedback loop between the cell and its microenvironment. Thus, a network of mRNA/miRNA/lncRNA is urgently required for rendering a comprehensive horizon in the mechanical mechanism of OTM and revealing the potential therapeutics.

There is an urgent need for more research notwithstanding recent advancements. ① Extensive lncRNAs have not been thoroughly explored, in contrast to coding RNAs and miRNAs, and their processes and roles are still unclear. It has been widely acknowledged that various lncRNAs play significant roles in bone remodeling. However, only PCR was used in the majority of research to confirm the expression of a number of genes following microarray or RNA-seq. Thus, further in-depth studies are warranted to explore the regulation of the identified lncRNAs. ② In addition, delivery strategies for miRNA therapy need to be investigated. Exosomes were found in the gingival crevicular fluid, and miR-29, for instance, showed enhanced expression after six weeks of OTM in humans, suggesting a possible regulatory role for miR-29 and a potential exosome-carried miRNA therapeutic system [160]. Considering that the biological functions of exosomes were confirmed, but exosome-based miRNA treatment remains elusive, more relative studies are deemed a promising outlook.

### Clinical implications

#### Accelerating orthodontic tooth movement (AOTM)

The rate-limiting phases that control the rate of OTM are believed to involve compression-generated osteoclast OC activity and bone-resorbing activity [17]. Therefore, efforts to speed up OTM should concentrate on bone resorption and osteoclast OC activity, two possible targets implicated in OC regulation that may aid in regulating OTM rates. For instance, local sclerostin protein injection on the compressed side of the alveolar bone in rats enhanced OTM by promoting osteoclastogenesis [161]. Still to be discovered are other significant regulators that mediate this network, and the therapy may benefit from their use.

Surgical techniques for accelerated orthodontic treatment have been studied in clinical practice for over 100 years [162], involving the original approaches, alveolar osteotomy alone or in combination with corticotomy, and modern approaches, selective alveolar corticotomy, which has become the gold standard for repeatability. Wilcko et al. [163] firstly proposed that rapid OTM after corticotomy may be due to a demineralization–remineralization process, which produces a regional acceleratory phenomenon of bone remodeling. Although surgical methods for quickening OTM are successful, physicians and patients have traditionally preferred non-surgical methods since they are less intrusive. These methods span the delivery of biomolecules topically or systemically to cutting-edge physical stimulation methods, all of which have demonstrated positive outcomes with various degrees of efficacy.

Due to their being noninvasive and painless, physical stimulation methods such resonance vibration, magnetic-filed forces, cyclic forces, mild electrical currents, low-intensity laser irradiation, and photobiomodulation are becoming more prevalent popular with patients and orthodontists. Low-level laser therapy increased the levels of IL-1β and MMP-9 and decreased the levels of MMP-8 in gingival crevicular fluid, which may induce the proliferation of OBs and OCs and lead to the process of alveolar bone remodeling [164]. Vibration promotes OC proliferation through NF-κB activation [165] and upregulates the PGE2 and RANKL in the PDLCs [166]. But vibration is also a controversial method, as some studies have shown that vibration had no impact on the acceleration of OTM [167, 168]. There is weak evidence that vibration stimulation is effective, which means that high-quality clinical trials and more scientific evidence are needed [169].

Extracellular vesicles (EVs) are small vesicles that provide a protective environment for the transport of various functional molecules. Current research [170] has demonstrated that EVs containing miRNAs are extensively involved in the communication among various cells during bone remodeling. MiRNAs could act as the hallmarks and therapeutic targets, while EVs could serve as the specific mail carriers, making them both biomarkers and therapeutics for bone disease.

#### Prevention of orthodontically induced inflammatory root resorption (OIIRR)

Orthodontically induced inflammatory root resorption (OIIRR) is an iatrogenic disorder that occurs during orthodontic treatment, in which the apical portion of the resorbed root is replaced by normal bone. Excessive root resorption is observed in 1–5% of orthodontic patients, with a loss of more than 4 mm or a third of the original root length [171]. The cause of OIIRR is unknown, but it is thought to involve a complex inflammatory process including multiple factors, such as MFs, root morphology, alveolar bone, PDL, cementum, and certain known biological messengers.

Pharmacological, minimally invasive, and noninvasive methods have been attempted to reduce root resorption. Makrygiannakis et al. [172] found that, compared with the control group, the root absorption of vitamin C-treated animals increased, while the application of alendronate, ibuprofen, growth hormone, low-dose meloxicam, simvastatin, lithium chloride, strontium ranelate attenuated the degree of root absorption. Likewise, Kaklamanos and colleagues [173] revealed that nabumetone administration reduces OIIRR, whereas fluoride was hardly observed to exert any effect. However, in another study, the administration of fluoride, thyroxine, and steroids decreased OIIRR [174]. Moreover, Amaro et al. [175] reported that the absence of estrogen after OTM induced OIIRR via imbalance of RANKL/OPG system, inflammatory, and osteoclastic makers in all strains of the mouse, indicating estrogen physiologically protects dental roots from orthodontic-induced inflammatory resorption. Besides, corticotomy and corticision used to accelerate OTM reduced root resorption in the early stages of OTM but aggravated root resorption with greater force [176]. Due to the risk of bias, indirect evidence, inconsistency heterogeneity, imprecision, and publication bias, the quality of the available evidence is considered low quality, and thus, high-quality clinical trials are required. Additionally, how to specifically control odontoblasts and cementum cells remains to be resolved. There is a tremendous effort underway to discover innovative medicines for the treatment of refined and accelerated OTM.

## Conclusion

**Fig. 6:**
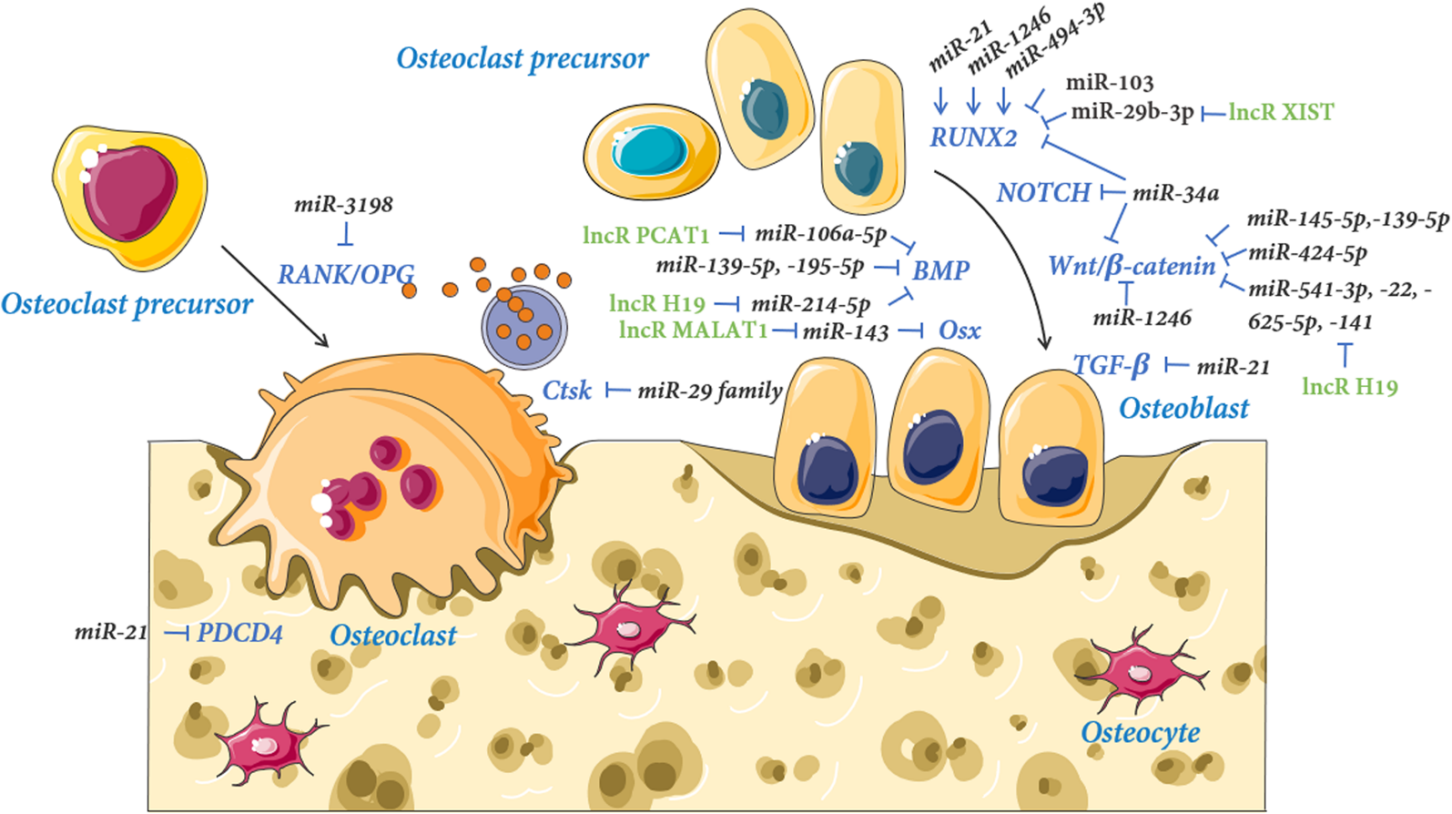
Mechano-sensitive ncRNAs in bone remodeling. The microRNAs and lncRNAs are regulated by various mechanical forces via altering gene targets. Obviously, the current research focuses on osteoblastic cell lines, and the mechano-sensitive RNA of osteoclast cell lines needs further studies

OTM is induced by MFs and is promoted by the remodeling of PDL and alveolar bone. The process of bone remodeling during OTM harbors distinctive mechanical features, mainly associated with tensive strain and compress stress. Previous research on the processes of tooth movement has concentrated on the essential signal pathways that drive the process. NcRNAs have captured the interest of the scientific community, particularly in the realm of personalized medicine, thanks to recent improvements in high-throughput sequencing analysis. Increasing studies demonstrate that multiple miRNAs and lncRNAs are essential for the regulation of several vital processes at every stage of mechanical response and bone remodeling (Fig. 6). And potentially regression, as well as modification of miRNA and lncRNA expression, could help to facilitate and accelerate alveolar bone remodeling during OTM.

Subsets of ncRNAs function during bone remodeling to control the progressive differentiation by inhibiting pathways that impede OB and OC differentiation at various time scales and in different cell types. It is evident that ncRNAs block both positive and negative transcriptional and cytokine regulators that function in intricate lncRNA-miRNA-mRNA-dependent networks, having both direct and significant indirect impacts on phenotypic formation. Further research is needed to understand how the various ncRNAs involved in the regulation of bone remodeling and formation coordinate the actions of these pathways.

Accelerating orthodontics and reducing complications during orthodontic procedures have long been the subject of extensive attention. But as we mentioned in “Clinical implications” section of the manuscript, there are exact limitations to the application of surgical techniques, physical stimulation techniques, and existing drugs. And from the existing theoretical framework, these three approaches perform poorly in addressing specificity issues. Therefore, from the perspective of precise regulation of bone remodeling, after extensive reading and discussion, ncRNAs, especially force-sensitive ncRNAs may become a breakthrough point for precise regulation of bone remodeling due to their important functional regulatory roles.

It is undeniable that the current research on ncRNA is not sufficient, and the evidence for clinical application is even less. Fortunately, research on targeting ncRNAs is accelerating and is widely discussed in [177–179]. In the field of orthodontics, targeting ncRNA therapeutic strategies lies in the following three parts: First, a network of mRNA/miRNA/lncRNA is urgently required for rendering a comprehensive horizon in the mechanical mechanism of OTM and revealing the potential therapeutics. Furthermore, the target ncRNA should be highly correlated and specific for OTM accelerating and root resorption. Finally, the delivery systems of ncRNA-targeted drugs, such as EVs, require further study. Based on the above discussion, this article will probably provide new ideas for orthodontic treatment and promote the research and application of ncRNA in bone remodeling. In summary, ncRNAs robustly regulate several aspects of osteogenesis and osteoclastogenesis, especially the MF-induced ones. Although numerous miRNAs and lncRNAs have been verified, only a few of them have been investigated for their association with tension and compression. Further advancements in the field of ncRNAs research will not only lead to the improvement of bone regeneration and a better understanding of the OTM process but also provide improved therapeutic candidates for improving the OTM treatment.

## Data Availability

Not applicable.

## Abbreviations

RANKL: Receptor activator of nuclear factor-kappa-Β ligand
OC-STAMP: Osteoclast-stimulatory transmembrane protein
PRISMA: Preferred Reporting Items for Systematic Reviews and Meta-Analyses statement
TRPV: Transient receptor potential vanilloid
INM: Inner nuclear membrane
SUN: Sad1/UNC-84
LINC: Linker of the nucleoskeleton and cytoskeleton
DKK1: Dickkopf-related protein 1
LRP: LDL-Receptor-related Protein
hBMSCs: Human bone marrow mesenchymal stem cells
HES1: Hairy and enhancer of split 1
ERK: Extracellular regulated protein kinases
MAPKs: Mitogen-activated protein kinases
RhoA: Ras homolog gene family member A
ROCK: Rho-associated protein kinase
YAP: Yes-associated protein
TAZ: Transcriptional co-activator with PDZ-binding motif
RT-qPCR: Real-time quantitative PCR
3’UTR: 3’-Untranslated region
5’-AZA: 5’-AZA-2’-deoxycytidine
GSK3β: Glycogen synthase kinase 3 beta
LDHA: Lactate dehydrogenase-A
JAG1: Jagged canonical Notch ligand 1
MMP: Matrix metalloproteinases
Hmga2: High mobility group AT-hook 2
Axin2: Axis inhibition protein 2
SORBS1: Sorbin and SH3 domain containing 1
WNT3A: WNT family member 3A
FGFR2: Fibroblast growth factor receptor 2
BMPR1A: Bone morphogenetic protein receptor-1A
SEMA3A: Semaphorin3A
ACVR2B: Activin receptor 2B
Smad 7: Smad Family Member 7
OPG: Osteoprotegerin
PDCD-4: Programmed cell death-4
FasL: Fas ligand
Cdc42: Cell division cycle 42
Srgap2: SLIT-ROBO Rho GTPase-activating protein 2
Gpr85: G protein-coupled receptor 85
Nfia: Nuclear factor I/A
Cd93: Complement component 1q receptor 1
ALP: Alkaline phosphatase
CNR2: Cannabinoid receptor 2
ceRNA: Competing endogenous RNA
ASCs: Adipose-derived stem cells
LCoR: Ligand-dependent corepressor
MAFB: V-maf musculoaponeurotic fibrosarcoma oncogene homolog B
Nedd4: Neural Precursor Cell Expressed Developmentally Down-Regulated Protein 4
